# Atypical Presentation of Scarlet Fever

**DOI:** 10.7759/cureus.33142

**Published:** 2022-12-30

**Authors:** Albandari Alotaibi, Maha A Binsaqr, May R Mutlaq, Asmaa A Khojah, Sahal A Khojah, Hind A Mohamed

**Affiliations:** 1 Family Medicine, Ministry of Health, Qassim, SAU; 2 College of Medicine, Sulaiman Al Rajhi University, Qassim, SAU; 3 Family Medicine, King Fahd Armed Forces Hospital, Jeddah, SAU; 4 Internal Medicine, International Medical Center, Jeddah, SAU

**Keywords:** rash, tonsillopharyngitis, streptococcus pyogenes, viral exanthem, scarlet fever

## Abstract

Scarlet fever is an infectious illness, which is caused by *Streptococcus pyogenes*. It causes exanthema and a characteristic tonsillopharyngitis. Its diagnosis is typically straightforward. However, due to the diverse clinical presentation of scarlet fever, one has to be cautious about atypical rash distribution that might go unrecognized or be misdiagnosed. Despite the fact that scarlet fever is primarily a pediatric illness, it can affect people of any age group. The case presented describes the clinical difficulty in the diagnosis of scarlet fever in an adult patient with atypical rash distribution involving dorsum of the hand and feet only till the level of wrists and ankles joints. A high degree of suspicion is required to diagnose this rare presentation and early treatment is essential to limit the spread of the disease.

## Introduction

Scarlet fever, also known as scarlatina, is a contiguous disease that affects the respiratory system. The disease is caused group A beta hemolytic streptococcus (GABS), and mainly contracted through droplet or direct contact with the skin or secretions of infected persons [1]. All age groups can be affected by the disease. However, children between 5 to 15 years of age are most commonly affected [1]. The condition is typically presented as acute pharyngitis, fever, and fatigue, which are followed by a sandpaper-like rash all over the body. However, some papers report other atypical clinical presentations [2]. Early identification and treatment with penicillin are essential to decrease the risk of complications, which range from mild to life-threatening complications [3].

## Case presentation

**Figure 1:**
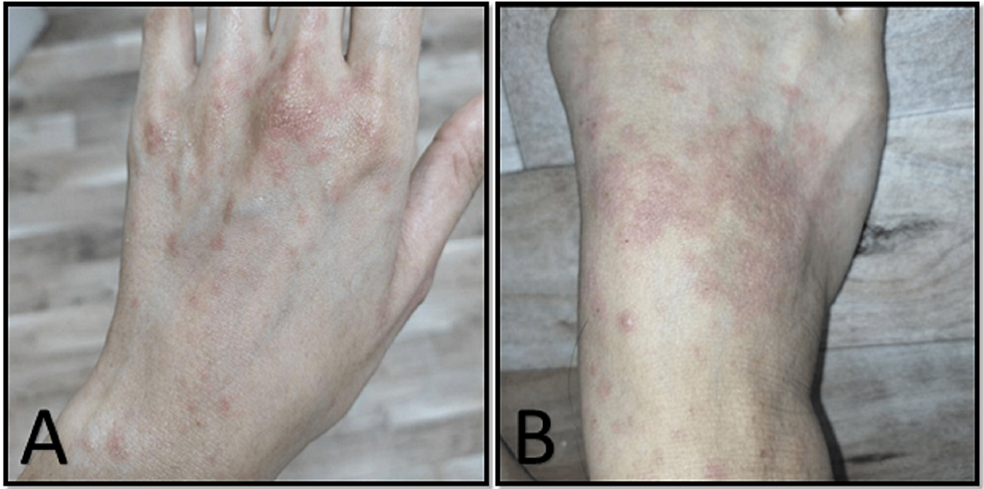
Multiple, blanchable erythematous macules covering the dorsal aspect of hands (A) and feet (B)

A 27-year-old girl attended the dermatology clinic for evaluation of skin rash. The rash started two weeks prior as asymptomatic red spots scattered on the dorsum of the hands and feet only. She denied any history of fever, sore throat, or other respiratory symptoms. There was no recent contact with ill patients and review of other systems revealed no complaints. On physical examination, the girl looked well and vitally stable. She had multiple blanchable erythematous macules covering the dorsal aspect of hands and feet till the level of wrists and ankles joints, respectively (Figure 1).

**Figure 2:**
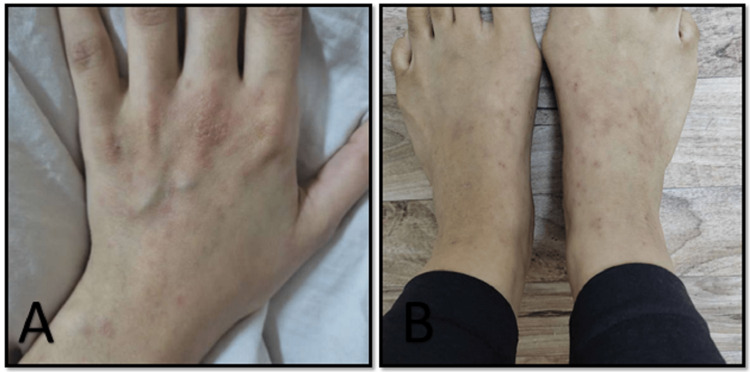
Improvement of the rash in hands (A) and feet (B) after starting the treatment

The patient's palms, soles, and rest of the body were clear with no petechiae or papules. Examination of the mouth revealed strawberry tongue with normal oral mucosa. No rash was detected at the hard or soft palate, and the cervical lymph nodes were not enlarged. Investigations showed normal complete blood count (CBC); Epstein Barr virus (EBV) markers and Viral Capsid Antigen (VCA) Immunoglobulin M (IgM) were negative. Antistreptolysin O (ASO) titer was 240 IU/ml. The diagnosis of scarlet fever was made and the patient prescribed amoxicillin-clavulanic acid, 625 mg twice daily for 10 days. The rash disappeared completely within a week (Figure 2). The differential diagnosis was papular purpuric gloves socks syndrome and other infectious rash like kawasaki.

## Discussion

Scarlet fever is caused by an infection of GABS. GABS is categorized into more than 200 types. Different strains of these bacteria have been implicated in the incidence of scarlet fever. A study from Brouwer et al. has shown that the most prevalent strains that are associated with scartlina were emm3, emm12, emm1, and emm4 [4]. Emm1 was characterized by severe invasive disease frequently.

Over the past century, a decline in the incidence of scarlet fever was seen in high income countries, but in recent years, the number of cases have been rising in the UK, mainland China, Hong Kong, and East Asia [4]. Patients affected by scarlet fever usually present with exudative pharyngitis and fever. A blanchable maculopapular skin rash typically starts 24 hours after the beginning of the condition: It appears first in the head then spreads to the other body parts. The rash is usually described as sandpaper rash due to the rough sensation of the skin caused by the raised pumps. Skin desquamation may appear as the rash starts to fade involving the palms and soles mostly. The tongue of the infected person might appear edematous and bright red, which is known as strawberry tongue [5]. The typical form of scarlet fever is not difficult to identify. However, there are uncommon presentations and non-obvious features that call for a differential diagnosis with other illnesses. A study conducted by Romero et al. found that 21 patients out of 91 had atypical presentations [6]. Those with uncommon presentation had smooth macular exanthem; plantaropalmar exanthema; erythema and swelling in the ear lobes; extensive erythroderma; localized facial erythroderma with coarse exanthema in the rest of the body; swelling of the eyelids, face, or the distal extremities; petechiae in the face, neck, and axillae; perianal dermatitis; and hives. All those divergent presentations were confirmed by microbiological testing as a scarlet fever [6]. Another similar but rare condition to scarlet fever is staphylococcal scarlet fever (SSF). Very limited reported cases are available regarding this condition. SSF is characterized by fever, malaise, and maculopapular exanthematous rash over the skin that is similar to scarlatina and intensified in flexures, later on, evolving into desquamation but without exfoliation. However, there is no enanthem over the mucus membranes or strawberry tongue as in scarlet fever caused by GABS. SSF is rare in general and especially in elderly population with only one paper reporting a SSF in a 74-year-old man [7]. Scarlet fever may also be mistaken for other illnesses, such as viral infections, Kawasaki disease, or toxic shock, due to its diverse clinical presentation. Microbiological testing such as rapid antigen detection test (RADT) or throat culture can help in confirming the diagnosis in these ambiguous cases. Scarlet fever can lead to suppurative complications caused by localized or hematogenous spread including; peritonsillar and retropharyngeal abscesses, cervical lymphadenitis, bacteremia, endocarditis, pneumonia, and meningitis. Other non-suppurative complications include rheumatic fever and glomerulonephritis [2]. Early identification of the disease and prompt treatment are necessary to avoid the unfavorable complication. The preferred treatment of scarlet fever is beta lactam antibiotics, penicillin V, or amoxicillin for 10 days, due to their approved cost effectiveness. Other options, if a patient is allergic to penicillin, include macrolides and cephalosporins [8].

## Conclusions

Scarlet fever is an infectious skin condition that can infect patient regardless of their ages. It present with skin rash that has a variable clinical pictures. Although rare, rash distribution over the dorsum of the hand and feet only is one of the atypical presentations that was not reported in the literature before. Physicians need to be cognizant of the possibility of countering patient with divergent pictures that can perplex the accuracy of the diagnosis. Careful history and full dermatological examination by health care practitioners may aid identification and timely treatment.
